# Marijuana Compounds: A Nonconventional Approach to Parkinson's Disease Therapy

**DOI:** 10.1155/2016/1279042

**Published:** 2016-12-05

**Authors:** Mariana Babayeva, Haregewein Assefa, Paramita Basu, Sanjeda Chumki, Zvi Loewy

**Affiliations:** Touro College of Pharmacy, 230 West 125th Street, Room 530, New York, NY 10027, USA

## Abstract

Parkinson's disease (PD), a neurodegenerative disorder, is the second most common neurological illness in United States. Neurologically, it is characterized by the selective degeneration of a unique population of cells, the nigrostriatal dopamine neurons. The current treatment is symptomatic and mainly involves replacement of dopamine deficiency. This therapy improves only motor symptoms of Parkinson's disease and is associated with a number of adverse effects including dyskinesia. Therefore, there is unmet need for more comprehensive approach in the management of PD. Cannabis and related compounds have created significant research interest as a promising therapy in neurodegenerative and movement disorders. In this review we examine the potential benefits of medical marijuana and related compounds in the treatment of both motor and nonmotor symptoms as well as in slowing the progression of the disease. The potential for cannabis to enhance the quality of life of Parkinson's patients is explored.

## 1. Introduction

Marijuana, the crude product (dried flowers, stems, seeds, and leaves) derived from the cannabis sativa plant, consists of more than 85 phytocannabinoids [1, 2]. The term phytocannabinoids is used to differentiate these plant-derived cannabinoids from the synthetic cannabinoids and the structurally different endogenous cannabinoids (endocannabinoids). Among the phytocannabinoids, Cannabidiol (CBD) and Δ9-Tetrahydrocannabinol (Δ9-THC, THC) are the major constituents of marijuana [3]. Δ9-THC is a psychoactive agent with analgesic and muscle relaxant property [3, 4]. While CBD is a nonpsychoactive compound and has been shown to have hypnotic, anxiolytic, antipsychotic, antioxidant, and neuroprotective effects [5], THC is a partial agonist at the cannabinoid receptor 1 (CB1) and receptor 2 (CB2). Unlike Δ9-THC, CBD has antagonistic/inverse agonistic property at CB1 receptor and appears to modulate Δ9-THC-associated side effects including anxiety, tachycardia, and hunger [3]. CBD also appears to potentiate the effect of endocannabinoids by inhibiting their inactivation, thereby alleviating psychotic symptom [6].

Despite the placement of marijuana in the schedule 1 category under the US Federal Controlled Substance Act [7] and the US Federal Government's continued opposition on its legalization, 24 states and Washington DC have enacted laws allowing marijuana to treat certain medical conditions [8]. The range and types of disease conditions for which medical marijuana have been approved vary from state to state. The most common disease conditions approved by the states include cancer, HIV/AIDS, glaucoma, chronic and/or severe pain, seizure/epilepsy, cachexia, and multiple sclerosis. Moreover, two cannabinoids (dronabinol and nabilone) have been approved by the FDA for clinical use. The synthetically produced Δ9-THC, dronabinol (Marinol®), is a schedule III drug, which is indicated in the treatment of chemotherapy-induced nausea and emesis as well as anorexia associated with weight loss in AIDS patients. A synthetic cannabinoid, nabilone (Cesamet®), is a schedule II drug that is indicated for the treatment of nausea and vomiting associated with cancer chemotherapy. Another cannabinoid, Cannabidiol (Epidiolex®), is in a clinical trial for the treatment of drug- resistant epilepsy in children [9]. A phytocannabinoid preparation, nabiximols (Sativex®), has been approved for the treatment of spasticity due to multiple sclerosis in a number of countries outside the United States. Nabiximols is an extract of* Cannabis sativa* L that consists of mainly THC and CBD [10, 11].

Although recent studies have provided strong evidence for the therapeutic benefit of medical marijuana [12–16], increasing access to cannabis and/or cannabinoids can result in side effects such as addiction, respiratory illness, and decline in cognitive processing. Cannabis use has been indicated as a potential cause, aggravator, or masker of major psychiatric symptoms, including psychotic, depressive, and anxiety disorders, particularly in young people [17–19]. Other negative effects include working memory deficits, reduced attention and processing speed, anhedonia, abnormal social behavior, and susceptibility to mood and anxiety disorders [20, 21]. While adult users seem comparatively resistant to cannabis-induced behavioral and brain morphologic changes, the individuals who start using cannabis during their early teens can have more severe and more long-lasting effects [22].

The target of medical marijuana and its constituents is the endocannabinoid system, which is involved in the modulation of a number of physiological functions. The endocannabinoid system includes the endocannabinoids, the cannabinoid receptors, and the enzymes involved in the biosynthesis and inactivation of the endocannabinoids [23] The cannabinoid receptors are mainly expressed in the central nervous system and the immune system, but they have also been identified in a number of other parts of the body including the cardiovascular system, the peripheral nervous system, the reproductive system, and the gastrointestinal tract. Due to its wide distribution and effects on a range of biological process, the cannabinoid system has become an attractive target for the development of drugs that can potentially be used for the treatment of a number of pathological conditions including mood disorders and movement disorders such as PD [24]. Components of the endocannabinoid system are abundant in the striatum and other parts of the basal ganglia and play a crucial role in modulating dopamine activity and motor functions [25–27].

Parkinson's disease (PD) is the second most common neurodegenerative disorder following Alzheimer's disease and the 14th leading cause of death in all age groups in the United States [28]. The prevalence of PD increases with age and is shown to be higher in males than females in some age groups [29]. The number of people with PD is projected at approximately 9 million by 2030 in the 15 most populous countries in the world [30, 31]. Neurologically PD is characterized by the destruction of dopaminergic cells in the pars compacta region of the substantia nigra in the midbrain, resulting in dopamine deficiency in the nerve terminals of the striatum in the forebrain [32]. These changes cause impairments not just to the motor system but also to the cognitive and neuropsychological systems [33]. The nigrostriatal pathway is one of the dopamine pathways in the brain that regulates movement. The exact cause for the loss of neuronal cells is unknown, and the trigger of dopaminergic degeneration seems to be multifactorial including environmental factors and genetic susceptibilities [34–36]. Clinically, PD is characterized by resting tremor, muscle rigidity, bradykinesia, and postural instability [32, 34, 37, 38] and it is also associated with a number of nonmotor symptoms including depression, anxiety, constipation, orthostatic hypotension, fatigue, and sleep disorders, as well as, in advanced disease, dementia [39–44]. Although dopamine deficiency accounts for the major motor symptoms of the disease, loss of noradrenergic and serotoninergic nerve terminals in the limbic system may account for several of the nonmotor features seen in Parkinson's disease [45, 46].

Current therapy involves treatment of motor symptoms of PD through replacement of dopamine deficiency [47]. This includes (1) enhancement of the synthesis of brain dopamine by administration of levodopa, a dopamine precursor, (2) direct stimulation of dopamine receptors, (3) decreasing dopamine catabolism, and (4) stimulation of dopamine release and inhibition of dopamine reuptake from presynaptic sites. Another therapy involves restoring the normal balance of cholinergic and dopaminergic actions on the basal ganglia using anticholinergic drugs [47–49].

However these drugs treat only motor symptoms of Parkinson's disease and are associated with a number of adverse effects. Long-term use of levodopa, the mainstay therapy for PD, is associated with motor fluctuations [50] and levodopa-induced dyskinesia [51–53]. The monoamine oxidase B (MAO-B) inhibitors (selegiline and rasagiline) as well as inhibitors of catechol-o-methyltransferase, COMT (tolcapone and entacapone), are used mostly to reduce the motor fluctuations associated with levodopa therapy due to their levodopa-sparing effect [54–59]. Several dopamine agonists including pramipexole, ropinirole, rotigotine, and apomorphine are used as monotherapy in early stage of Parkinson disease or as adjunctive therapy with levodopa in patients with advanced PD in order to reduce motor fluctuations [56, 60–64]. In addition to their limited efficacy on motor symptoms and their adverse effects, drugs that are currently used for the treatment of PD do not have an effect on disease progression. Therefore, there is an urgent need for the development of safer drugs that treat both the motor and nonmotor symptoms of PD as well as drugs that slow the progression of the disease.

Medical marijuana has been demonstrated to improve motor symptoms including tremor, rigidity, and bradykinesia as well as nonmotor symptoms such as pain and sleep disorders of PD in observational studies [65]. Survey of PD patients in Colorado, USA, also indicated the beneficial effects of marijuana in alleviating nonmotor symptoms of PD [66]. Cannabidiol (CBD), one of the major constituents of marijuana, has been shown to be effective in the treatment of psychosis and sleep disorders in PD patients [67–69]. Another phytocannabinoid, Δ9-tetrahydrocannabivarin (Δ9-THCV, THCV), was studied in animal disease model of PD and found to have neuroprotective and symptom-relieving effects [70]. Therefore, marijuana may provide an alternative or add-on therapy for Parkinson's disease. In addition, Parkinson's disease has been listed as one of the disease conditions for which medical marijuana is allowed in Connecticut, Illinois, Massachusetts, New Hampshire, New Mexico, and New York. However, it may also be covered under chronic illnesses in several other states.

In this review we seek to investigate any scientific evidence that indicates the potential use of marijuana and/or its components for the treatment of Parkinson's disease. The review aims to (i) examine briefly current treatment and the unmet need of PD therapy, (ii) assess the role of the cannabinoid system in the modulation of movement and neuroprotection, (iii) look at the mechanism of action of marihuana constituents in the modulation of movement and PD-associated disorders, (iv) assess other beneficial effects of marihuana that contribute to the amelioration of PD, and (v) gather scientific evidence on the clinical benefit of marijuana and/or its constituents in PD patients.

## 2. Marijuana and Its Influence on the Endocannabinoid System

Cannabis has been used to treat disease since ancient times. Marijuana is derived from the* Cannabis sativa L. *plant. Marijuana contains the active chemicals known as cannabinoids. At least 85 cannabinoids have been identified as unique compounds in* Cannabis *[1]. The therapeutic potential of many of these ligands still remains largely unexplored prompting a need for further research. The chemicals responsible for the medicinal effects of marijuana are D9-Tetrahydrocannabinol (THC) and Cannabidiol (CBD) [71, 72]. THC is the major psychoactive ingredient, acting primarily upon the central nervous system where it affects brain function. CBD is the major nonpsychoactive ingredient in cannabis and produces neuroprotective and anti-inflammatory effects [73]. Both compounds, TCH and CBD, have anticonvulsant properties [74]. Cannabinoids have also potential to alleviate motor disorders by reducing motor impairments and neuron degeneration [75]. In addition, cannabinoids have been shown to be effective in preclinical studies involving excitotoxicity, oxidative stress, neuroinflammation, and motor complications associated with PD [76].

Some cannabinoids (endocannabinoids or ECBs) are found in the body. Initially, ECBs were discovered in the brain and subsequently in the periphery in humans and animals. Endocannabinoids are produced by cultured neurons [77], microglia, and astrocytes [78]. ECBs interact with the endocannabinoid system and aid in regulation of memory, pleasure, concentration, thinking, movement and coordination, sensory and time perception, appetite, and pain [24, 79, 80]. The ECBs activate two guanine nucleotide-binding protein- (G-protein-) coupled cell membrane receptors, consequently named the cannabinoid type 1 (CB1) and type 2 (CB2) receptors [81]. CB1 receptors are located primarily in the central and peripheral neurons and CB2 receptors are predominantly found in immune cells [82]. CB1 receptors are important mediators in signaling pathways and have been identified on both glutamatergic and gamma*-*aminobutyric (GABA) neurons [83]. It is believed that one important role of the neuronal CB1 component is to modulate neurotransmitter release in a manner that maintains homeostasis by preventing the development of excessive neuronal activity in the central nervous system [82]. Animal models illustrate that activation of the CB1 receptor by their endogenous ligands can result in prominent neuroprotective effects and may prevent epileptic seizures [84]. Other studies suggest that activation of CB1 receptors offers neuroprotection against dopaminergic lesion and the development of L-DOPA-induced dyskinesias [85]. CB2 receptors are closely related to CB1 and are mainly expressed on T cells of the immune system, on macrophages and B cells, and in hematopoietic cells [86]. They are also expressed on peripheral nerve terminals where these receptors play a role in antinociception and the relief of pain [87]. In the brain, CB2 receptors are mainly expressed by microglial cells, where their role remains unclear [88].

The major identified ECBs are arachidonoyl ethanolamide (anandamide, AEA), 2-arachidonoyl glycerol (2-AG), O-arachidonoyl ethanolamine (virodhamine), and 2-arachidonoyl glyceryl ether (noladin ether) [89]. Both AEA and 2-AG are specific ligands of CB1 and CB2 receptors. Besides having activity on CB1 and CB2 receptors, AEA also has full agonistic activity at TRPV1 receptor [90]. AEA is localized in the brain and periphery [91]. In the brain AEA shows high distribution in the hippocampus, thalamus, striatum, and brainstem and to a lesser extent in the cerebral cortex and cerebellum [92]. Lower concentrations of AEA are found in human serum, plasma, and cerebrospinal fluid [93]. Similarly, 2-AG is observed in both the brain and periphery, although its concentration is almost 150 times higher in brain compared to that of AEA [92, 94, 95]. 2-AG has greater potency, stability, and agonistic activity at CB1 and CB2 receptors compared to that of AEA [96, 97]. Two prominent areas involved in the control of movement, such as the globus pallidus and the substantia nigra, contain not only the highest densities of CB1 receptors [88], but also the highest levels of ECBs, especially AEA [98, 99]. Tissue levels of AEA are regulated by fatty acid amide hydrolase (FAAH) [100]. It has also been shown that the basal ganglia contain the precursor of AEA [98, 99], supporting the theory of in situ synthesis for this compound. Studies have demonstrated that AEA synthesis is regulated by dopaminergic D2 receptors in the striatum, suggesting that the endocannabinoid system acts as an inhibitory feedback mechanism countering the dopamine-induced facilitation of motor activity [101].

Marijuana compound THC is CB_1_ and CB_2_ receptor partial agonist [82]. Due to the structural similarity of natural cannabinoid THC to the endogenous cannabinoid AEA, many therapeutic advantages of THC have been identified, such as lowering ocular pressure, inhibiting smooth muscle contractions, and increasing appetite [102]. When smoked, THC is rapidly absorbed from the lungs into the bloodstream and has an effect on the cannabinoid receptors. The central nervous system and specific areas of the brain contain the highest concentration of cannabinoid receptors. Therefore, cannabis or THC administration can create an overexcitation of the system that results in altered perceptions, pleasure, and mood [103].

Unlike THC, CBD has little affinity for CB1 and CB2 receptors but acts as an indirect antagonist of cannabinoid agonists. While this should cause CBD to reduce the effects of THC, it may potentiate THC's effects by increasing CB1 receptor density or through another CB1-related mechanism [73]. CBD is also an inverse agonist of CB2 receptors. CBD can counteract some of the functional consequences of CB1 activation in the brain, possibly by indirect enhancement of adenosine A1 receptors activity through equilibrative nucleoside transporter (ENT) inhibition [73]. CBD helps to augment some of THC's beneficial effects, as it reduces the psychoactivity of THC, enhances its tolerability, and widens THC's therapeutic window [104].

Other cannabinoids can also contribute to the cannabis medicinal effects. Studies in experimental models and humans have suggested anti-inflammatory, neuroprotective, anxiolytic, and antipsychotic properties of chemicals extracted from marijuana [6, 15, 82, 105, 106].

## 3. Cannabinoids and Parkinson's Disease

### 3.1. Changes in the Cannabinoid System in Parkinson's Disease

Recent data from several studies indicate the important role of the endocannabinoid system in Parkinson's disease. The components of the endocannabinoid system are highly expressed in the neural circuit of basal ganglia, which is part of a complex neuronal system. This neuronal system coordinates activities from different cortical regions that directly or indirectly participate in the control of movement [107, 108]. In the basal ganglia, the endocannabinoid system bidirectionally interacts with dopaminergic, glutamatergic, and GABAergic signaling systems [109]. Endocannabinoids play a dominant role in controlling transmission at synapses between cortical and striatal neurons, in mediating the induction of a particular form of synaptic plasticity, and in modulating basal ganglia activity and motor functions [110]. The progressive loss of dopaminergic neurons that occurs in PD leads to lower striatal levels of dopamine. These low levels of dopamine result in the alteration of the equilibrium between the direct and the indirect basal ganglia pathways and ECB signaling [111].

The cannabinoid signaling system mentioned above experiences a biphasic pattern of changes during the progression of PD [112]. Early and presymptomatic PD stages, characterized by neuronal malfunction with little evidence of neuronal death, are associated with desensitization/downregulation of CB1 receptors and aggravation of various cytotoxic insults such as excitotoxicity, oxidative stress, and glial activation [113]. However, intermediate and advanced stages of PD, characterized by a deep nigral degeneration and manifestation of major Parkinsonian symptoms, are associated with upregulatory responses of CB1 receptors and the endocannabinoid ligands [113]. This could explain the potential of CB1 receptor ligands in alleviating common PD symptoms.

In the brain, CB1 receptors are expressed by GABAergic neurons innervating the external and internal segments of the globus pallidus and the substantia nigra [114–116]. CB1 receptors are also present in the corticostriatal glutamatergic terminals and in the excitatory projections from the subthalamic nucleus to the internal segment of the globus pallidus and the substantia nigra [114–116]. Within the striatum, CB1 receptors are expressed in parvalbumin immune-reactive interneurons, cholinergic interneurons, and nitric oxide synthase-positive neurons [117, 118]. Animal models of Parkinson's disease show an increase in the density of CB1 receptors, levels of endogenous ligands, and CB1 receptor binding in the basal ganglia [119–122]. Endogenous cannabinoids activate CB1 receptors on presynaptic axons and reduce neurotransmitter and glutamate release, working as retrograde synaptic messengers released from postsynaptic neurons [123]. Similarly, activation of CB1 receptors inhibits both glutamate release from substantia nigra afferents and GABA release from striatal afferents. At the same time, activation of presynaptic CB1 receptors in the external segments of the globus pallidus can increase local GABA levels by reducing GABA reuptake from striatal afferents to the nucleus and decrease GABA release from striatal afferents of the substantia nigra [114, 116, 118]. Based on these evidences, it is thought that the function of the basal ganglia neuronal system is controlled by ECB. The presence of endocannabinoid systems in different neural structures and their interaction with dopaminergic, glutamatergic, and GABAergic neurotransmitter signaling systems make the components of endocannabinoid system ideal targets for a novel nondopaminergic treatment of PD.

Endocannabinoid signaling is also bidirectionally linked to dopaminergic signaling within the basal ganglia [118]. The CB1, D1, and D2 dopamine receptors are localized in the striatum [114, 115]. In animal models, CB1 and D2 dopamine receptors share a common pool of G proteins, suggesting the link of their signal transduction mechanisms [124, 125]. In addition, D2 receptor stimulation resulted in release of ECBs in the striatum [101]. However, stimulation of CB1 receptors completely inhibited D1-dopamine receptor mediated activation of adenylyl cyclase and decreased GABA release from striatal afferents of dopaminergic neurons of the substantia nigra resulting in an increased firing of these cells [114–116].

Another receptor involved in control of movement is transient receptor potential vanilloid type 1 (TRPV1), which is expressed in sensory neurons and basal ganglia circuitry of dopaminergic neurons [126, 127]. TRPV1 receptors are molecular integrators of nociceptive stimuli activated by endovanilloids [128]. TRPV1 also interacts with ECB. In particular, anandamide is one of the major endogenous activators of TRPV1 [129–131]. Studies have revealed that motor behavior can be suppressed by the activation of vanilloid receptors [98, 99], suggesting that TRPV1 receptors might play a role in the control of motor function.

### 3.2. Preclinical Data on the Endocannabinoid System as a Target for Parkinson's Disease Therapy

The association of cannabinoids with regulation of motor functions is well established [132–135]. The effect of the cannabinoids on motor activity depends on the impact of the endocannabinoid system on the dopaminergic, glutamatergic, and GABAergic signaling systems throughout the basal ganglia [112, 136]. The high density of cannabinoid, dopamine, and vanilloid-like receptors coupled with ECBs within the basal ganglia and cerebellum suggests a potential therapeutic role for the cannabinoids in the control of voluntary movement and in movement disorders such as Parkinson's disease [98, 99, 121, 137]. Additional indications of an important role of the endocannabinoid system in the control of movement involve an inhibitory action of cannabinoids through fine tuning of various classical neurotransmitters activity [138], prominent changes in transmission of ECBs in the basal ganglia [139], and alteration of the CB1 binding as well as CB1 availability in the substantia nigra [85, 112, 119, 120, 140, 141]. These data support the idea that cannabinoid- based compounds act on vital pathways of endocannabinoid transmission and therefore might be of therapeutic interest due to their potential to diminish motor symptoms in extrapyramidal disorders such as Parkinson's disease [27, 76, 142].

Research with cannabinoid agonists and antagonists demonstrates that the cannabinoids can modulate motor activity and produce alterations in corresponding molecular correlates [129, 143–145]. It has been widely reported that synthetic, plant-derived, or endogenous cannabinoid agonists exert a powerful motor inhibition in laboratory species [129, 144, 146–149]. This hypokinetic effect was shown to be mediated by the activation of CB1 receptors in neurons of the basal ganglia circuitry [88, 137, 141, 150–152]. Stimulation of the CB1 receptor by a synthetic cannabinoid HU-210 decreased spontaneous glutamatergic activity and reduced the rotations induced by levodopa/carbidopa by 34% in PD rats [153, 154]. Administration of CB1 receptor agonists THC and two synthetic cannabinoids WIN 55,212-2 and CP 55,940 increased extracellular dopamine concentrations in rats [152, 155, 156]. WIN 55,212-2 and CP 55,940 also weakened contralateral rotations induced by a selective D_1_/D_5_ receptor partial agonist SKF38393 without developing catalepsy in PD rats [148]. In a gender study THC produced an increase in tyrosine hydroxylase activity in parkin-null male mice (a model of early stages of PD) and caused a motor inhibition that was significantly greater compared to wild-type animals [122]. Treatment with THC inhibited motor activity and produced catalepsy in rats [109, 144, 146, 147] and caused antinociception and ring immobility in mice [157]. In other studies THC diminished the motor inhibition caused by 6-hydroxydopamine [70] and potentiated the hypokinetic effect of reserpine in rats more than 20-fold [135]. However, in a primate model of Parkinson's disease THC did not affect locomotor activity but increased bradykinesia [125].

Administration of WIN 55,212-2 increased stimulation of GTP*γ*_S_ binding in the caudate nucleus, putamen, globus pallidus, and substantia nigra of marmosets, indicating an effective activation of CB1 signaling mechanisms [119, 120]. WIN 55,212-2 produced a dose-dependent reduction of the spontaneous motor activity and catalepsy in mutant Syrian hamsters, increased antidystonic efficacy of benzodiazepines [158], and significantly reduced the antikinetic effects of quinpirole in the reserpine-treated rats [159]. Treatment with WIN 55,212-2 also reduced levodopa-induced dyskinesias, attenuated axial, limb, and severe orolingual abnormal involuntary movements in 6-hyroxydopamine- (6-OHDA-) lesioned rats [160–163]. An endogenous cannabinoid agonist oleoylethanolamide (OAE) produced reduction in dyskinetic contralateral rotations correlated with reduction of molecular associates of L-DOPA-induced dyskinesia: reduced FosB striatal overexpression and phosphoacetylation of hystone 3 [164]. Another synthetic agonist levonantradol decreased general and locomotor activity and increased bradykinesia in a primate model of Parkinson's disease [125]. Nabilone, a synthetic cannabinoid agonist, coadministered with levodopa significantly decreased total dyskinesia compared with levodopa alone treatment and increased the duration of antiparkinsonian action of levodopa by 76% in PD marmosets [165, 166].

Cannabinoid agonist anandamide (AEA) and its synthetic analog methanandamide increased the extracellular dopamine levels in the nucleus accumbens shell of rats by the activation of the mesolimbic dopaminergic system [167]. This dopamine increase was inhibited by the cannabinoid CB1 receptor antagonist rimonabant [167]. However, recent discoveries indicate that AEA is also able to activate vanilloid VR(1) receptors and that the activation of these receptors might also be responsible for changes in nigrostriatal dopaminergic activity and anandamide-induced hypokinesia [168–170]. AEA produced a tonic facilitation of glutamate release in the substantia nigra via stimulation of VR1 receptors, indicating the involvement of this receptor in motor and cognitive functions of the dopaminergic system [171]. Preclinical data have shown that AEA decreased the activity of nigrostriatal dopaminergic neurons and produced hypokinesia that was completely reversed by an antagonist of vanilloid-like receptors, capsazepine [129]. Additional studies have demonstrated that AEA inhibited ambulation and stereotypic behavior, increased inactivity, and occluded the effects of an agonist of vanilloid VR_1_ receptors, livanil, on locomotion in mice, suggesting a common mechanism of action for the two compounds [170]. Treatment with anandamide lowered motor activity with the maximal inhibition by approximately 85% and produced hypothermia and analgesia in mice, increased the inactivity time, and markedly decreased the ambulation and the frequency of spontaneous non-ambulatory activities in rats [146, 147, 172, 173]. Moreover, AEA produced a decrease in spontaneous motor activity in laboratory animals similar to the reported actions of THC [129, 145, 153, 170]. The hypokinetic actions of AEA were boosted by coadministration with a selective inhibitor of endocannabinoid uptake N-(3-furylmethyl) eicosa-5,8,11,14-tetraenamide, UCM707 [174].

Tissue concentrations of endocannabinoids are important for producing motor effects. Levels and activities of AEA and 2-AG can be manipulated by inhibition of FAAH enzyme, the action of which is reduced in experimental models of PD [153, 175]. Animal studies have shown that the FAAH enzyme inhibitor [3-(3-carbamoylphenyl) phenyl] N- cyclohexylcarbamate (URB597) magnified and prolonged a rapid, brief dopamine increase that was produced by AEA [167]. Additional studies have confirmed that FAAH inhibition remarkably increases AEA tissue levels but reduces 2-AG levels [176, 177]. To determine whether FAAH inhibition has beneficial impact on PD symptoms the effect of the FAAH inhibitor, URB597, was studied in MPTP- lesioned marmosets. Treatment with URB597 increased plasma levels of AEA, did not modify the antiparkinsonian actions of L-DOPA, and reduced the magnitude of hyperactivity to levels equivalent to those seen in normal animals [178]. In PD mice URB597 prevented induced motor impairment [179]. Moreover, other FAAH inhibitors, JNJ1661010 and TCF2, also have anticataleptic properties [179]. These results reveal that FAAH inhibition may represent a new strategy for treatment of PD.

Overall, these results indicate that endogenous or exogenous cannabinoid agonists activate the dopaminergic system and play a very important role in modulation of motor behavior [180]. In addition to the effects on movement activity, cannabinoid agonists have demonstrated neuroprotective properties, suggesting that the cannabinoids have a promising pharmacological profile for not only improving Parkinsonian symptoms but also delaying PD progression [70, 85, 181–183].

The CB1 receptor antagonists can also influence movement syndromes of Parkinson's disease suggesting that modulation of the CB1 signaling system might be valuable in treatment of motor disorders. In a study with PD rats rimonabant (SR141716A), a selective antagonist of CB1 receptors has shown the potential to act as an antihypokinetic agent by enhancing glutamate release from excitatory afferents to the striatum [184]. Moreover, SR141716A prevented the effects of THC on dopamine release [156, 167] and also increased the locomotor activity in mice and rats preexposed to THC [170, 185]. SR141716A produced a 71% increase in motor activity in MPTP-lesioned marmosets with LID [136]. Coadministration of SR141716A with levodopa resulted in significantly less dyskinesia than administration of levodopa alone [136, 160]. SR141716A also reversed effect of the cannabinoid agonist WIN 55,212-2 and increased the locomotor activity in 6-OHDA-lesioned animals [159, 163]. Coadministration of SR141716A with a selective D_2_/D_3_ receptor agonist quinpirole reduced levels of AEA and 2AG by sevenfold in the globus pallidus, boosted the locomotive effects of quinpirole, and produced restoration of locomotion in animal models of Parkinson's disease [98, 99, 101, 136, 186]. In parkin-null mice SR141716A produced a decrease in tyrosine hydroxylase activity in the caudate–putamen and as result formed a hyperkinetic response [122]. However, SR141716A did not alleviate the motor deficits in a primate model of Parkinson's disease [125].

Another CB1 receptor antagonist AM251 and SR141716A produced antiparkinsonian effects in rats with very severe nigral degeneration (>95% cell loss) [187]. Local administration of these antagonists into denervated striatum, globus pallidus, and subthalamic nucleus reduced motor asymmetry in Parkinsonian rats [187, 188], which was inhibited by CB1 receptor agonist AM404 [187]. Another CB1 antagonist CE-178253 produced a 30% increase in motor behavior responses to L-DOPA in MPTP-treated rhesus monkeys but did not modify levodopa-induced dyskinesias [189]. THCV caused changes in glutamatergic transmission and attenuated the motor inhibition in PD rats [70]. Overall, these findings suggest that cannabinoid CB1 antagonists might be therapeutically effective in the control of Parkinson's disease and levodopa-induced dyskinesia [114, 190].

The activation of CB2 receptors might also contribute to some extent to the potential of cannabinoids in PD [191]. THCV, which is not only a CB1 antagonist but also a CB2 partial agonist, reduced the loss of tyrosine hydroxylase-positive neurons in the substantia nigra with preservation of these neurons in CB2 receptor-deficient mice [70]. CBD has also reduced the loss of tyrosine hydroxylase-positive neurons in the substantia nigra of PD rats. Both compounds, THCV and CBD, have acted via neuroprotective and antioxidant mechanisms [70, 182, 191]. CBD has also demonstrated significant effects in preclinical models of neurodegenerative disorders in combination with other cannabinoids [15, 70, 192]. CB2 receptor agonists display a promising pharmacological profile for delaying disease progression.

The cannabinoid pharmacologic manipulation represents a promising therapy to alleviate movement disorders and levodopa-induced dyskinesias. Thus, CB1 antagonists appear to have antiparkinsonian effects, while cannabinoid receptor agonists may be useful in the treatment of motor complications in Parkinson's disease.

### 3.3. Effect of Cannabinoids on Patients with Movement Disorders

Cannabis and related compounds have created significant research interest as a promising therapy in neurodegenerative and movement disorders. The successful use of tincture of* Cannabis indica *in treating PD was first described in Europe by Gowers [193]. Despite the lack of controlled studies, there is evidence that cannabinoids are of therapeutic value in the treatment of tics in Tourette syndrome, some forms of tremor and dystonia, chorea in Huntington's disease, the reduction of levodopa-induced dyskinesia in Parkinson's disease, and Parkinsonian syndromes [194–201].

A study with smoked cannabis queried 339 PD patients indicated that marijuana produced significant improvement of general PD symptoms in 46% of the patients; 31 % of them reported improvement in resting tremor, 38% reported relief from rigidity, 45% defined reduced bradykinesia, and 14% of the patients reported alleviated dyskinesias [202]. High urine concentration (>50 ng/ml) of the THC primary active metabolite, 11-HO-THC, was associated with relief from PD symptoms [202]. The dose and frequency of the cannabis administrations were important in relieving PD symptoms. Smoked cannabis also produced a statistically significant improvement in tremor, rigidity, and bradykinesia as well as improvement in sleep and pain scores in 22 PD patients [65]. In another study, smoked cannabis was responsible for a significant improvement in the mean total motor Unified Parkinson's Disease Rating Scale (UPDRS) score, tremor, rigidity, and bradykinesia in 17 patients with PD [203]. One dose of smoked marijuana provided symptoms relief for up to 3 hours [203]. Moreover, both studies reported significant improvement of nonmotor symptoms of PD, such as pain and sleep [65, 203]. However, smoked marijuana did not reduce Parkinsonian symptoms in 5 patients with idiopathic Parkinson's disease and severe tremor [204]. A clinical trial in 19 PD and 6 patients with levodopa-induced dyskinesia demonstrated that oral cannabis extract was ineffective for alleviating parkinsonism or dyskinesia [205].

Few studies have evaluated the effects of CBD on PD symptoms. In a pilot study CBD lowered total UPDRS scores and significantly reduced psychotic symptoms in 6 PD patients with psychosis [67]. In another study CBD administration produced no improvement in measures of motor and general symptoms in 21 PD patients [68, 69]. However, the group treated with CBD had significantly different mean total scores in the Parkinson's Disease Questionnaire, 39 compared to the placebo group [68, 69]. Oral CBD improved dyskinesia by up to 30% without a significant worsening of the parkinsonism in PD patients [206]. CBD withdrawal caused severe generalized dystonia [206].

Clinical studies have been conducted to evaluate the effect of a synthetic cannabinoid nabilone. Oral nabilone significantly reduced dyskinesia without aggravating parkinsonism in seven PD patients with severe L-DOPA-induced dyskinesia [207]. In another study, nabilone produced a 22% reduction in levodopa-induced dyskinesia in PD patients [208]. Nabilone showed efficacy not only against LID but also against bradykinesia in PD patients [209]. Some other cannabinoid related compounds such as CE178253, OEA, and HU-210 have also been reported to be efficacious against L-DOPA-induced dyskinesia and bradykinesia in PD [199, 209]. However, SR 141716 did not improve Parkinsonian motor disability in PD patients [210]. The American Academy of Neurology (AAN) review deemed marijuana “probably ineffective” for treating L-DOPA-induced dyskinesia [211]. These conflicting results indicate the need for more research in this area.

Several clinical studies have been performed to evaluate the effect of marijuana on dystonia. Inhaled cannabis has provided a marked reduction in dystonia and complete pain relief in patients with right hemiplegic painful dystonia. Moreover, the patients have been able to completely discontinue opioid use [212]. Smoked cannabis also improved idiopathic dystonia and generalized dystonia due to Wilson's disease [213, 214]. In a preliminary study, administration of CBD resulted in a 50% improvement in spasm severity and frequency in a patient with blepharospasm-oromandibular dystonia [215] and amelioration of the dystonic movements within 2-3 hours in patients with dystonic movement disorders [201]. CBD also improved dystonia by 20–50% in dystonic patients and stopped tremor and hypokinesia in 2 patients with Parkinson's disease [200]. Another cannabis compound, THC, produced a reduction of abnormal movement patterns in a 14-year-old girl with marked dystonia [216] and decreased intensity of myoclonic movements in a 13-year-old boy with athetosis and myoclonic movements [216]. In contrast to these findings, one study found no significant reduction in dystonia following treatment with nabilone [165, 166].

Studies have looked at the potential benefits of medical marijuana and cannabinoids for the treatment of Huntington's disease (HD). Nabilone versus placebo showed a treatment difference of 0.86 for total motor score; 1.68 for chorea; 3.57 for Unified Huntington's Disease Rating Scale (UHDRS) cognition; 4.01 for UHDRS behavior; and 6.43 for the neuropsychiatric inventory in HD patients [217]. However, in previous study nabilone was found to increase choreatic movements in patients with HD [197, 198]. AAN guideline examining the efficacy of marijuana for treating chorea in HD stated nabilone can be used for modest decreases in HD chorea [218]. Available data regarding the effect of CBD on HD symptoms are inconsistent. CBD produced improvement (20–40%) in the choreic movements in HD patients [219]. However, a latter study did not confirm the earlier finding [220]. A comparison of the effects of CBD and placebo on chorea severity in neuroleptic-free HD patients indicated no significant or clinically important differences [220].

Few studies have indicated that marijuana and THC can reduce tics and associated behavioral disorders in patients with Tourette's syndrome (TS) [221]. Cannabis inhalations produced a significant amelioration of TS symptoms [222]. Following marijuana administration 82% of TS patients (*N* = 64) reported a reduction, or complete remission of motor and vocal tics, and an amelioration of premonitory urges and obsessive-compulsive symptoms (OCB) [199]. Smoked marijuana also eliminated TS symptoms in one case study [223]. Administration of THC to a boy with TS improved tics and enhanced short-interval intracortical inhibition and the prolongation of the cortical silent period [224]. TCH significantly reduced tics and improve driving ability in a Tourette's patient [225]. Treatment with THC lowered the mean C1 specific over nonspecific binding ratio (*V*_3_′′) from 0.30 to 0.25 in six TS patients, although the difference was not significant. However *V*_3_′′ clearly declined in a patient with a marked clinical response [226]. To date, there have been only two controlled trials that investigated the effect of THC on TS [194], both of which reported a significant improvement of tics and OCB after THC administration [195, 196].

Considering the relevance of these data, the need for alternative treatments for PD motor and nonmotor symptoms, medical marijuana, or related compounds may provide a new approach to the treatment of Parkinson's disease.

## 4. Beneficial Effects of Cannabinoids in the Amelioration of Nonmotor Symptoms and Progression of Parkinson's Disease

### 4.1. Neuroprotective Actions of Cannabinoids

Cannabinoids have been shown to have neuroprotective effect due to their antioxidative, anti-inflammatory actions and their ability to suppress exitotoxicity. Plant-derived cannabinoids such as THC and CBD can provide neuroprotection against the in vivo and in vitro toxicity of 6-hydroxydopamine and this was thought to be due to their antioxidative property or modulation of glial cell function or a combination of both [182]. Studies found that CBD was able to recover 6-hydroxydopamine-induced dopamine depletion and also induced upregulation of Cu, Zn-superoxide dismutase, which is a key enzyme in endogenous defense against oxidative stress [70, 191, 227]. The reported data suggest that CBD also diminishes the increase in nicotinamide adenine dinucleotide phosphate (NADPH) oxidase expression and decreases the markers of oxidative stress, inflammation, and cell death in the kidneys [228]. Another study has also emphasized a role for superoxide anion produced by microglial NADPH oxidase in augmenting the demise of dopaminergic neurons in the PD brain [229]. The mechanism by which CBD acts to reduce NADPH oxidase expression and inhibit oxidative injury within the PD brain has yet to be confirmed but it seems to act through mechanisms independent of CB1 or CB2 receptors [76]. However, data obtained from recent studies have hinted towards a direct relationship between the CB1 receptor and mitochondrial functions in the brain [230]. The phenolic ring moieties in cannabinoids display antioxidant activity guarding against glutamate-induced neurotoxicity in a cellular model [231]. CBD produced reduction of hydroperoxide-induced oxidative damage and was more protective against glutamate neurotoxicity compared to ascorbate and a-tocopherol, indicating that CBD is a potent antioxidant [232]. Taken together, these discoveries support the hypothesis that treatment with cannabinoids having antioxidant effects may modulate mitochondrial reactive oxygen species production [233] in the PD brain.

Inflammation has been shown to be a crucial pathological factor responsible for the demise of dopaminergic neurons in PD [234–236]. Glial cells appear to play a key role in neuroinflammation, since higher levels of activated microglia are reported in the substantia nigra of patients with PD compared to brains of control subjects [237, 238]. Cannabinoids demonstrate anti-inflammatory activities by suppressing toxic cytokine release and microglia activation [181–183]. Increased CB2 receptor expression in nigral cells and stimulation of these receptors protect dopaminergic neurons from microglia-induced inflammation and regulate neuronal survival [70]. The cannabinoids are known to be able to activate the CB2 receptor, which mediate the anti-inflammatory effects of the compounds and preserve cells from excessive apoptosis. Recent evidence substantiates that some cannabinoids may attenuate the neuroinflammation associated with PD [191, 239–241]. Several studies showed that CBD has anti-inflammatory properties [242–246] and can produce beneficial effect in acute inflammation and chronic neuropathic states [5, 247, 248]. THC demonstrates anti-inflammatory effect via activation of the CB1 receptor [249–251]. In addition, cannabinoids provide anti-inflammation effect by reducing the vasoconstriction and restoring blood supply to the injured area [252]. All these data support that cannabinoids are potentially effective compounds for the treatment of neuroinflammatory conditions, including neurodegenerative diseases like PD.

Marijuana may prevent brain damage by protecting against neuronal injury. There are a few mechanisms by which cannabinoids provide neuroprotection. One of the mechanisms involves an induction/upregulation of cannabinoid CB2 receptors, mainly in reactive microglia, and regulates the influence of these glial cells on homeostasis of surrounding neurons [253]. In combination with the increased antitoxic effects observed in cell cultures containing glia, this suggests that immunomodulation produced by CB2 receptor activation may play a primary role in the neuroprotective properties of cannabinoids [182]. Another mechanism of neuroprotection is activation of CB1 receptors. Loss of dopaminergic neurons and greater degree of motor impairment in CB1 knockout mice have been reported [85]. Cannabinoids activating the CB1 receptor are antiexcitotoxic due to suppression of glutamatergic activity with a subsequent decrease in calcium ion influx and eventual nitric oxide production [254–256]. Sativex-like combination of phytocannabinoids has been demonstrated to produce neuroprotective effect via interaction with both CB1 and CB2 receptors [134, 257]. In addition, THC reduced the loss of tyrosine hydroxylase-positive neurons in the substantia nigra [70] and exhibited neuroprotective effect by activation of the PPAR*γ* receptors [258]. Overall, these data suggest that cannabinoids are neuroprotective in acute and chronic neurodegeneration and can delay or even stop progressive degeneration of brain dopaminergic system, a process that cannot be prevented currently.

### 4.2. Analgesic Effect of Cannabinoids

Pain is a relevant and often underestimated nonmotor symptom of PD [259, 260]. Pain affects more that 50% of people with this disorder and can cause extreme physical, psychological, and social disorders and worsen Parkinsonian disability [261, 262]. Different treatment options are used to treat PD pain [262–265]. However, these medications have significant side effects and do not provide universal efficacy [264, 265]. Cannabis is well known as a pain-relieving plant. The cannabinoid receptors in the central and peripheral nervous systems have been shown to modulate pain perception [266, 267].

Several clinical studies have been performed to investigate the effect of marijuana or cannabinoids on pain. Smoked cannabis significantly reduced neuropathic pain intensity as well as significantly improved mood disturbance, physical disability, and quality of life in HIV-patients [268]. Cannabis was effective at ameliorating neuropathic pain in patients with central and peripheral neuropathic pain [269]. Inhaled cannabis significantly reduced pain intensity (34%) compared to placebo in a clinical trial of painful distal symmetric polyneuropathy (DSPN) [270]. Whole plant extracts of* Cannabis sativa* produced statistically significant improvements on the mean pain severity score [271]. Cannabis-based medicine significantly decreased chronic pain intensity as well as sleep disturbance in multiple sclerosis patients [272, 273]. Oromucosal nabiximols (1 : 1 combination of the THC and CBD) produced a reduction in pain intensity scores in patients with neuropathic pain [274].

These findings are consistent with other discoveries supporting the efficacy of cannabis in relieving pain. The analgesic effect of cannabinoids has been reviewed [75, 211, 275–281]. The review of the literature suggests that marijuana and/or cannabinoids may be efficacious for pain relieving in various disease states including PD.

### 4.3. Antidepressant Effect of Cannabinoids

Depression is one of the common nonmotor symptoms of PD and the estimated rate varies widely, with an average prevalence of up to 50%. [282–284]. Despite its association with poor health outcomes and quality of life, depression in PD patients is underdiagnosed and undertreated [285–287]. Studies have indicated that the endocannabinoid system is involved in the regulation of mood and emotional behavior, and the loss or blockade of the endocannabinoid signaling system results in depressive symptoms [288]. For example, the CB1 receptor antagonist rimonabant has been shown to induce symptoms of anxiety and depression [289–291]. In addition, polymorphism of the gene that encodes the CB1 receptor has been associated with depression in PD [292]. In animal models, low level of THC produced antidepressant activity and increased serotogenic activity via activation of the CB1 receptor [293]. Animal studies have also shown that inhibition of hydrolysis of the endocannabinoid anandamide exerts antidepressive effect [294] and resulted in an increased serotonergic and noradrenergic neuronal activity in the midbrain. Currently available antidepressant drugs act via increasing serotonin and/or noradrenaline levels. These, and many other studies, indicate that the cannabinoid system is a potential target for the development of novel antidepressant drugs. Epidemiological studies have demonstrated that people who used cannabis daily or weekly exhibit less depressed mood and more positive effect than nonusers of cannabis [295]. Other studies have shown an association between heavy cannabis use and depressive symptoms. However, it is not clear whether the increased depressive symptoms are due to cannabis use or other factors that increased the risk of both depression and heavy use of cannabis [296]. Therefore, moderate use of cannabis in PD patients may help alleviate depressive symptoms and improve quality of life.

### 4.4. Effect of Cannabinoids on Sleep Disorders

Sleep disorders are common in PD patients and negatively affect the quality of life. The reported prevalence ranges from 25% to 98% and this wide variation could be due to differences in study design and diagnostic tools used [297]. The causes of the sleep disturbances in PD are multifactorial and include neurodegeneration and the medications used to treat motor symptoms of PD [298]. Various sleep disorders including rapid eye movement sleep behavior disorder, insomnia, sleep fragmentation, excessive daytime sleepiness, restless legs syndrome, and obstructive sleep apnea have been described in PD patients [299, 300]. Cannabidiol, the major nonpsychotic component of marijuana, has been reported to improve rapid eye movement sleep behavior disorder in PD patients [68, 69]. Marijuana has also been shown to improve nonmotor symptoms of PD including sleep [65]. In clinical trials involving 2000 patients with various pain conditions, nabiximols has been demonstrated to improve subjective sleep parameters [301]. Thus, marijuana could be used to enhance the quality of life of PD patients by alleviating sleep disorders and pain.

## 5. Summary

Cannabis and related compounds have recently been studied as promising therapeutic agents in treatment of neurodegenerative and movement disorders including Parkinson's disease. In this review we have examined the potential benefits of medical marijuana and cannabinoids in the treatment of both motor and nonmotor symptoms as well as in slowing the progression of the disease. We have looked into any scientific evidence that indicates the potential use of marijuana and/or related compounds for the treatment of PD. Current treatments of PD provide only relief of motor symptoms and are associated with adverse effects such as dyskinesia. In addition, these therapies do not slow the progression of the disease. Therefore, there is an urgent need for safer drugs that can treat both motor and nonmotor symptoms of PD as well as drugs that slow the progression of the disease.

In spite of the placement of marijuana in schedule 1 category under the US Federal Controlled Substance Act, 24 states and Washington DC have enacted laws allowing the use of marijuana to treat a range of medical conditions. Parkinson's disease has been listed as one of the disease conditions for which medical marijuana is allowed in a number of states. Research studies have provided evidence for the potential effectiveness of medical marijuana and its components in the treatment of PD as cannabinoids act on the same neurological pathway that is disrupted in Parkinson's disease. Involvement of the endocannabinoid system in the regulation of motor behavior, the localization of the cannabinoid receptors in areas that control movement, and the effect of cannabinoids on motor activity indicate that cannabinoids can be potentially used in the treatment of movement disorders. Cannabinoid agonists and antagonists have been shown to modulate the endocannabinoid system and modify motor activity. Cannabinoid receptor antagonists appear to produce antiparkinsonian effects while cannabinoid receptor agonists exert a powerful motor inhibition and may be useful in the treatment of motor complications. In addition, we have assessed the role of the cannabinoid system and marijuana constituents in neuroprotection as well as considered other beneficial effects of marijuana. Marijuana has been shown to improve nonmotor symptoms of PD such as depression, pain, sleep, and anxiety. Moreover, components of cannabis have been demonstrated to have neuroprotective effect due to their anti-inflammatory, antioxidative, and antiexcitotoxic properties. Due to combination of the above mentioned beneficial effects, cannabis may provide a viable alternative or addition to the current treatment of Parkinson's disease. However, there are concerns regarding the use of medical marijuana including lack of standardization and regulation, imprecise dosing, possible adverse effects, and medication interactions. Further studies are needed to provide more data on efficacy, safety, pharmacokinetics, and interactions of cannabinoids.
